# Evaluation of Pollution of Soils and Particulate Matter Around Metal Recycling Factories in Southwestern Nigeria

**DOI:** 10.5696/2156-9614-8.17.20

**Published:** 2018-03-12

**Authors:** Akinade S. Olatunji, Tesleem O. Kolawole, Moroof Oloruntola, Christina Günter

**Affiliations:** 1 Department of Geology, University of Ibadan; 2 Department of Geological Sciences, Osun State University, Osogbo, Osun State; 3 Department of Geosciences, University of Lagos, Akoka, Lagos; 4 Institute of Earth and Environmental Science, University of Potsdam, Germany

**Keywords:** potentially toxic elements, metal recycling plants, slags, pollution indices

## Abstract

**Background.:**

Metal recycling factories (MRFs) have developed rapidly in Nigeria as recycling policies have been increasingly embraced. These MRFs are point sources for introducing potentially toxic elements (PTEs) into environmental media.

**Objectives.:**

The aim of this study was to determine the constituents (elemental and mineralogy) of the wastes (slag and particulate matter, (PM)) and soils around the MRFs and to determine the level of pollution within the area.

**Methods.:**

Sixty samples (30 slag samples, 15 soil samples and 15 PM samples) were collected for this study. The soils, slag and PM samples were analyzed for elemental constituents using inductively coupled plasma optical emission spectrometry. Mineralogy of the PM was determined using scanning electron microscope-energy dispersive spectroscopy (SEM-EDS), and soil mineralogy was determined by an X-ray diffractometer (XRD).

**Results.:**

The results of the soil analyses revealed the following concentrations for the selected metals in mg/kg include lead (Pb) (21.0–2399.0), zinc (Zn) (56.0–4188.0), copper (Cu) (10.0–1470.0), nickel (Ni) (6.0–215.0), chromium (Cr) (921.0–1737.0) and cadmium (Cd) (below detectable limit (Bdl)-18.1). For the slags the results were Pb (68.0–.333.0), Zn (1364.0–3062), Cu (119.0–1470.0), Ni (12.0–675.0), Cr (297–1737) and Cd (Bdl-15.8). The results in μg/g for the metal analysis in PM were Pb (4.6–160.0), Zn (18.0–471.0), Cu (2.5–11.0), Ni (0.8–4.2), and Cr (2.5–11.0), while Cd was undetected. The slags are currently utilized for filling the foundations of buildings and roads, providing additional pathways for the introduction of PTEs into the environment from the suspended materials generated from mechanical breakdown of the slags.

**Conclusions.:**

The MRFs were found to have impacted the quality of environmental media through the introduction of PTEs, impairing soil quality, in addition to PM, which can have detrimental health consequences. Further studies on the health implications of these pollutants and their impacts on human health are needed.

**Competing Interests.:**

The authors declare no competing financial interests

## Introduction

Metals have many industrial applications and metal recycling has become increasingly attractive owing to the shrinking worldwide reserves of primary ore minerals, increased level of consumption and need to reduce and mitigate potentially toxic materials in the environment.^1^,^2^,^3^,^4^,^5^,^6^,^7^

However, the processes involved in metal recycling (sorting, dismantling, crushing and smelting) often result in the production of enormous amounts of waste materials such as slags, gaseous fumes and untreated waste water that are enriched with heavy metals. These metals pollute the air, soil, sediment, water bodies, and other environmental media, and thus there is a need for monitoring of the effects of metal recycling plants on their immediate environment.

Metal recycling has increased rapidly in the last decade owing to governmental measures banning the exportation of scrap metals. This has created incentives for investors and as a result unregulated metal recycling factories (MRFs) are being set up in Nigeria. These MRFs have become important economic enterprises, providing low-income jobs for thousands of people involved in the sorting and dismantling of scrap metal.

**Figure 1:**
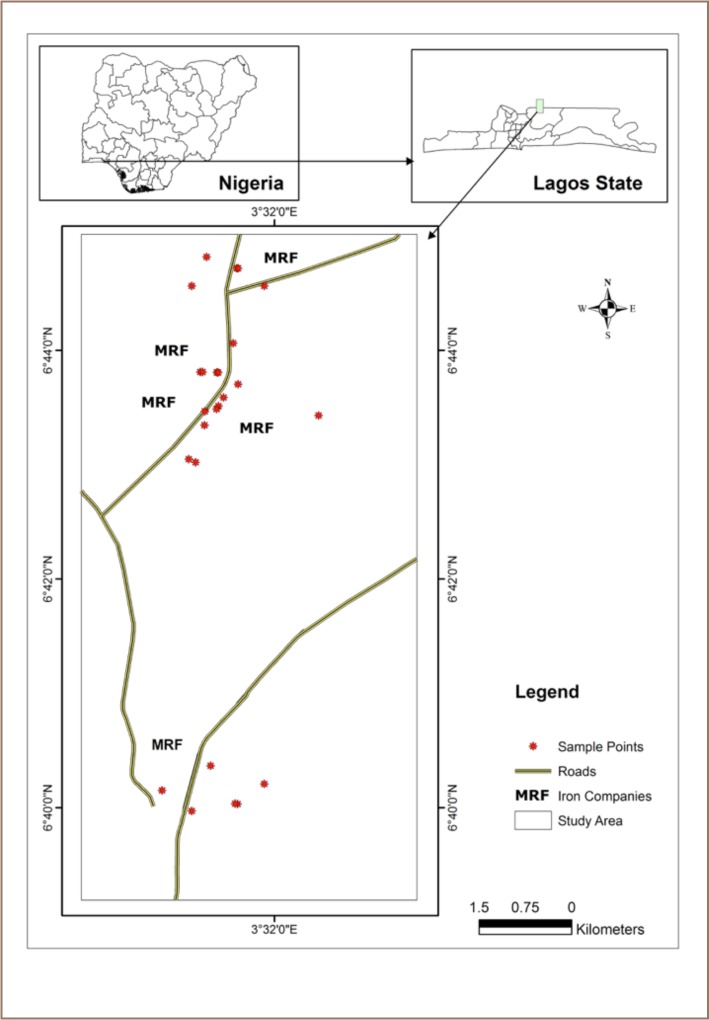
Map of the study area

**Figure 2:**
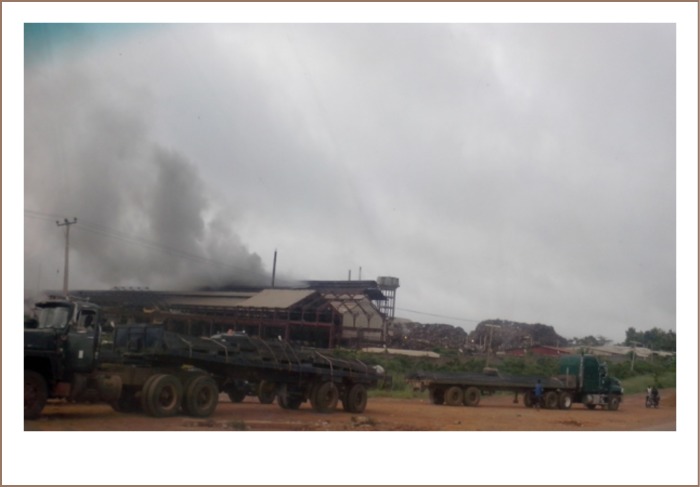
Fumes produced from one of the metal recycling factories

Metal recycling processes often result in the production of large amounts of waste materials such as slags, fumes and untreated waste water that are enriched in heavy metals which pollute the soil (*Figures 1 and 2*) and other environmental media.^2^,^3^,^6^

The MRFs surveyed in this study use electric arc furnaces in the production of steel from scrap metals. An electric arc furnace is a batch process with input materials of scrap metals and fluxing materials loaded into the cylindrical, refractory-lined furnace. The current passing through the graphite electrodes generates heat, which subsequently melts the scrap. These processes result in the generation of slag (a mixture of silicate materials with non-ferrous metals), large volumes of particulate matter emissions, and metal-laden wastewater. The emissions from MRFs are usually enriched with potentially toxic elements (PTE), silica and lime.^2^,^4^

Potential health problems due to exposure to these pollutants include increased allergy, asthma, cardiovascular and cardio-pulmonary diseases.^8–12^ Where exposure is prolonged, cases of various cancers have been reported.^8–12^ Some heavy metal pollutants have also been implicated in disorders of the nervous system.^9^,^11^

The chemical composition of particulate matter (PM) can vary widely as a function of emission source and the subsequent chemical reactions which take place in the atmosphere.^13^,^14^ Particulate matter has been associated with a wide range of illnesses including pulmonary and cardiovascular disorders.^15^,^16^,^17^,^18^ Human health is affected by PM when it is transported over densely populated areas,^19^ retained in residences and other occupied structures,^20^ and also impacts the nutrient loading of waters flowing from adjacent watersheds^21^ and terminal bodies of water by direct and indirect deposition.^22^,^23^

**Abbreviations**
*MRFs*: Metal Recycling Factories
SEM-EDS: Scanning Electron Microscope-Energy Dispersive Spectroscopy

Several studies on the effects of air pollution on health have indicated a strong positive correlation between air pollution concentration and observed health effects.^24^,^25^,^26^ Long-term exposure to air pollution PM increases the risk of lung cancer, respiratory diseases and arteriosclerosis, and short-term exposure can exacerbate several forms of respiratory diseases, including bronchitis and asthma, as well as cause changes in heart rate variability.^27^,^28^,^29^,^30^,^31^,^32^

Wastes associated with MRF activities are often dumped in the immediate surroundings. The siting of MRFs does not follow any defined pattern and they are situated in densely populated residential areas. The present study aimed to determine the heavy metal concentration of PTEs in the wastes of MRFs in Nigeria and associated environmental impacts.

## Methods

This investigation undertook a geochemical assessment of the metal concentration levels in slags, soils and PM around the MRFs in southwestern Nigeria.

### Study Area

**Figure 3:**
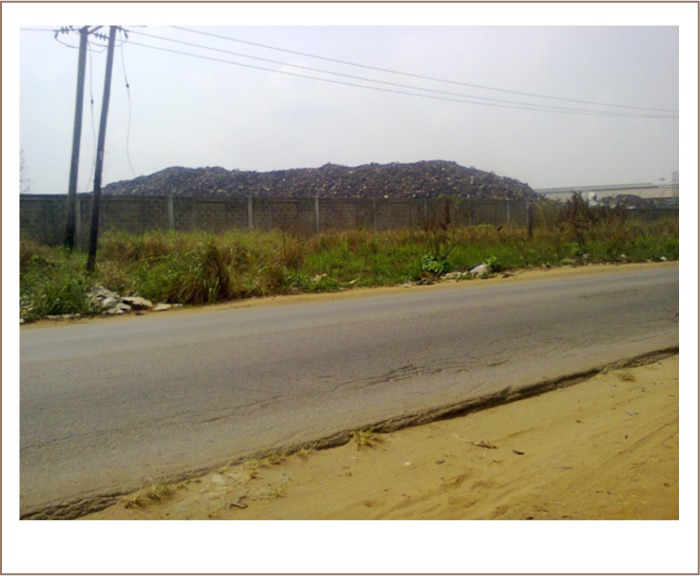
‘Scrap metal hill’ waste pile in a metal recycling factory

The study area lies within longitudes 3°31.277′ and 3° 22.386′ and latitudes 6° 39.970′ and 6° 44.717′ (*Figure 3*) The factories are well connected by accessible roads with the main Ikorodu-Sagamu highway running through the area. There are currently over 10 MRFs located within the axis with many more under construction. This area is home to arguably the largest conglomeration of MRFs in Nigeria, and possibly Africa. The MRFs receive hundreds of scrap metal-laden trucks from all over the country. These scrap metals are recycled into billets and iron rods. The MRFs stock pile scrap metals on their premises leading to generation of ‘scrap metal hills’ within their compounds (*Figures 2 and 3*).

**Figure 4:**
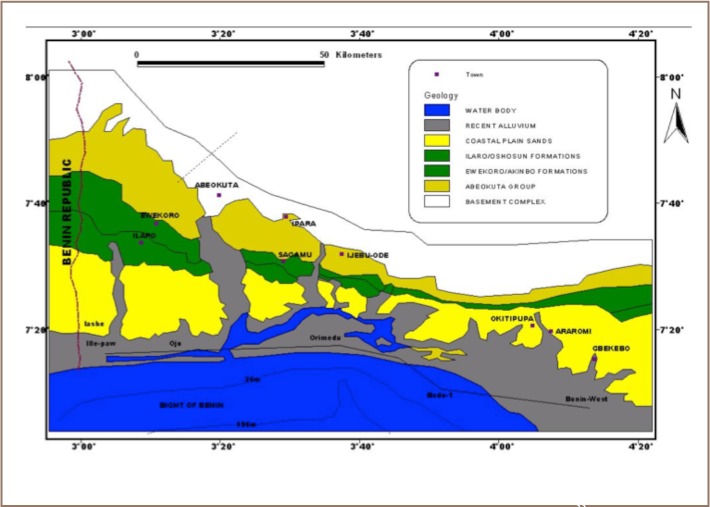
Geology of the Eastern Dahomey Basin^33^

Geologically, the area is underlain by Tertiary to Recent Coastal Plain Sands of southwestern Nigeria made up of a repetitive succession of clay and sandy horizons (*Figure 4*). The clay ranges from reddish-brown to dirty-white and the sands range from very fine to coarse and gravelly in texture. Minor occurrences of peat and ferruginous sandstones layers have also been reported in the area.^5^

### Sampling

Twenty-one sampling stations were selected around the MRFs on the basis of accessibility. At each sampling station, 4 soil samples (about 500 g each) were obtained from each station in a 10 m square grid. These were then made into one composite (about 2 kg) sample to represent that location. Sixty of the soil samples made into fifteen composites were eventually analyzed for the study.

The sampling stations were located on unoccupied plots and along roadsides. Soil samples were obtained from depths ranging from 0–15 cm using a plastic scoop. Plastic scoops were cleaned with cotton wools soaked in methanol after every location to prevent cross contamination. The soil samples were air-dried under room temperature, disaggregated and sieved to remove debris, dirt and plant roots. Particulate matter was sampled with a high volume air sampler (#15000, Science Source Company) capable of measuring particles under <10 μm in size. Particles were sampled on 120 mm diameter, 1 μm pore-size cellulose filters. The sampling duration was between 4 and 6 hours. The filters were weighed before and after collection with 0.001 mg precision using a digital Mettler balance (PL203, Mettler Toledo). This was done after preconditioning for 48 hours at constant humidity (40–42%) and temperature (20–22°C), in accordance with previous work.^34^ Thirty boulder-like slag samples (about 150 g) were collected randomly (grab sampling) from several slag dumps outside the MRFs and pulverized prior to elemental analysis.

The retrieved filter papers were divided into four equal parts and two portions of the four parts were digested in aqua regia. The digested samples were analyzed for constituent particles and elemental distribution using scanning electron microscope-energy dispersive spectroscopy (SEM-EDS). The results generated from the SEM/EDS analysis were subjected to size distribution analysis and data reduction techniques such as hierarchical cluster analysis that were employed in order to identify particles according to their chemical similarity^35^,^36^,^37^ using the IBM Statistical Package for the Social Sciences (SPSS 17, 2008).

For the elemental constituents, the PM filters, sieved soil samples (0.5 g) and pulverized slag samples (0.5 g) were digested in hot acid combination. This was achieved by adding 5 ml of nitric acid (Merck Suprapur 65%), 2 ml of hydrochloric acid (Merck Suprapur 36%) and 10 ml of ultra-pure water (18 MΩ cm^−1^ of specific resistivity) to a Pyrex tube and heated for 2 hours at 95°C on a heating plate.^18^ The extracted solution was then filtered, using a Whatman N°41 (WH1441-110) filter, completed to 50 ml with ultra-pure water and kept in pre-cleaned polyethylene bottles in the refrigerator until analyzed. Filter and reagent blanks were processed following the same treatment. All elemental analyses (soil, slag and PM) were performed using ICP-OES. The elemental analyses results were subjected to correlation and r-mode factor analyses to ascertain similarities and contrasts in chemical composition.

All sample locations were determined using the global positioning system (GPS). Soil samples mineralogy was determined using the X-ray diffractometer (XRD) method.

## Results

The selected metals for geochemical evaluation were Pb, Zn, Cu, Ni, Cr and Cd. The heavy metal results showed contrasting and varied concentrations in the soils, PM and slag samples (*Table 1, Supplemental Material 1*). A comparison of the metal contents of the soils from the study area with those from other industrial areas of the world, where similar environmental studies have been undertaken, revealed that the analyzed metals are in much higher concentrations in the study area (*Table 2*).

**Table 1:**
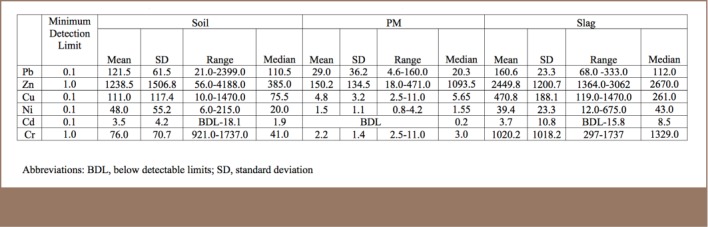
Statistical Summary of Geochemical Results of the Three Media in the Study Area

**Table 2:**
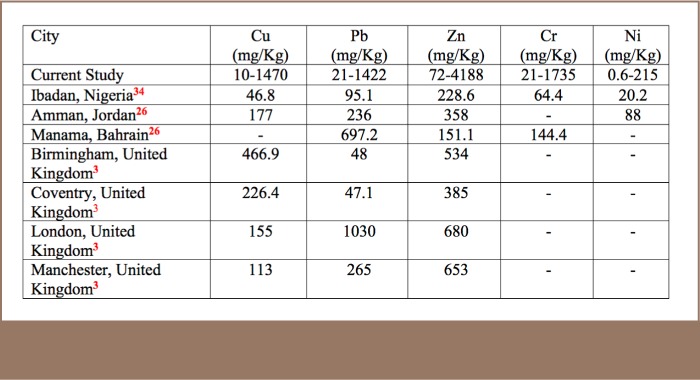
Comparison of Metal Contents of Soil Across Cities

### Particulate Matter

**Figure 5:**
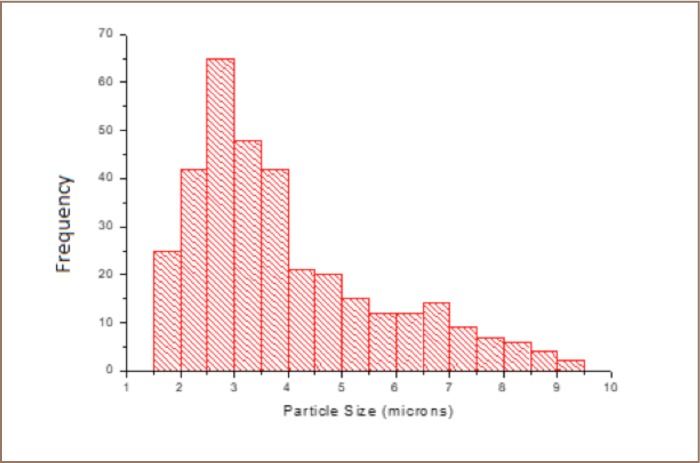
Particle size distribution of the airborne particulate matter in the study area

**Figure 6:**
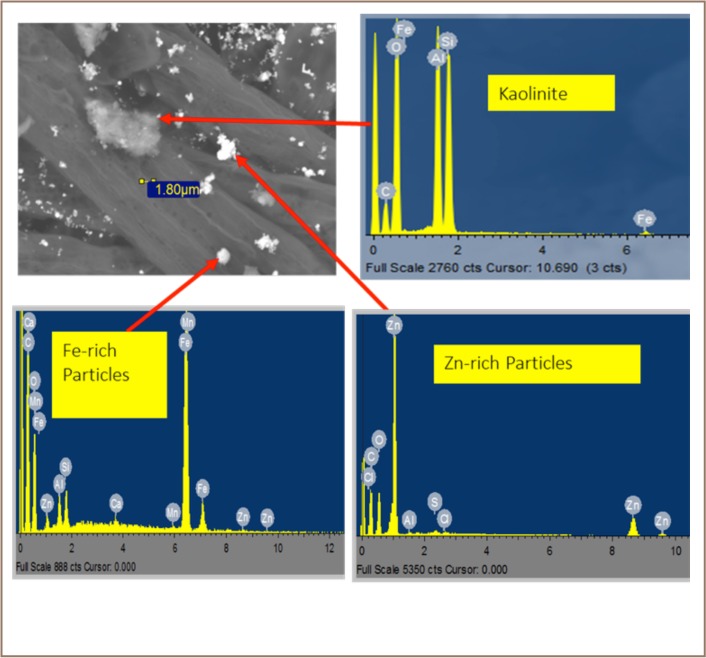
Backscattered image from SEM/EDS of particles in particulate matter

One thousand and five hundred (1500) particles were studied with the SEM-EDS. A majority of the particles were coarse and modally skewed between 2.5–4 μm, with some even larger (*Figure 5*). The mineralogy of PM (*Table 3* and *Figure 6*) samples is dominated by zinc-rich, iron-rich, and clay minerals particles including chlorites, kaolinite, illite, smectite, feldspars, quartz, sulfur + clay particles (*Figure 6 and Supplemental Material 2*).

**Table 3:**
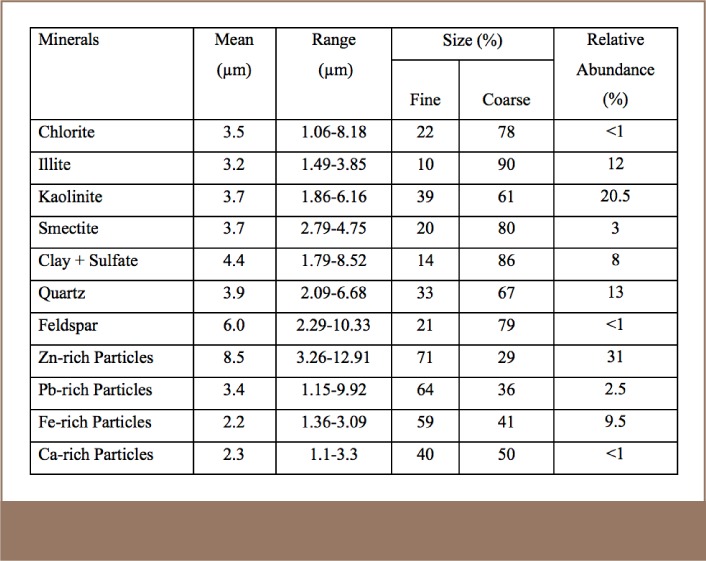
Description of the Size Range of Particulate Matter Particles

#### Zinc-rich Particles

Zinc-rich particles were the most abundant, accounting for around 31% of the PM. These are characterized by high Zn content and are mostly finely rounded to sub-rounded in shape. The coarse ones are mostly associated with clay minerals (*Figure 6*).

#### Iron-rich Particles

Iron-rich particles were next in abundance (9.5%), occurring as spherical to irregular coarse fractions ranging from 1.4–3.1 μm in size.

#### Quartz-rich Particles

Quartz particles are characterized by fine mode fractions associated with Al, Ca and Fe. It accounted for about 13% of PM content (*Table 3* and *Supplemental Material 2*).

#### Lead-rich Particles

Lead-rich particles are fine to coarse and make up about 2.5% of PM. The particles are associated with chlorine and zinc.^38^,^39^

#### Clay- and Sulfur-containing Particles

Kaolinite, illite, chlorite and smectite make up about 35.5% of PM. Clay particles have a pronounced irregular shape and are coarse in size. These particles contain mainly aluminum and silicon, with varying amounts of Mg, Ca, K and Fe. The clay particles originate from windblown soil dust and re-suspension of dust from unpaved roads in the area.

Around 8% of the clay rich particles contained sulfur, especially the smectite/illite group, whose surface was observed to be coated with sulfur.

#### Calcium-rich Particles

The sizes of the observed calcium-rich particles ranged from 1.1–3.3 μm, and they were rounded and generally fine. These particles were characterized by high content of Ca, moderate amounts of S and Si, and minor amounts of Mg, Al, Cl, K, and Fe (*Table 3* and *Supplemental Material 2*).

### Soil Characterization

The XRD analysis of the soils revealed quartz and kaolinite with trace amounts of anatase and hematite as the dominant minerals in the soil samples (*Supplemental Material 3*). The presence of the hematite confers a reddish-brown color to the lateritic soil.

## Discussion

### Nature of Particulate Matter and Soil

The presence of zinc in the PM was an indication the scraps contained zinc that was not recovered in the recycling process. This zinc could have resulted from recycled galvanized metal scraps (*Table 3* and *Supplemental Material 2*). Iron-rich particles are often associated with Zn and Mn and to some extent with alumino silicate particles. Inefficient smelting processes may have resulted in their incorporation in PM as informal waste scavengers often look through the discarded slags for unrecovered metals (*Table 3* and *Supplemental Material 2*). Quartz presence in PM could have resulted from the re-suspension of road particles and from quartzite used as one of the raw materials in the metal recycling plants. Sulfur is usually emitted into the atmosphere as SO_2_ from combustion of fossil fuel (in this case, diesel). The resulting chemical reactions in air form sulfate particles in the form of ammonium sulphate, which could interact with mineral components of atmospheric dust in various forms.^40^,^41^ Studies have shown that there is very low reactivity between sulphate and aluminosilicate minerals and therefore sulfate tends to coat the surface of aluminosilicate minerals.^42^,^43^,^44^

The abundance of quartz and kaolinite in the soils could also be the reason for the preponderance of quartz-rich and clay-rich particles in the studied PM.

### Environmental implications

To evaluate the impact of the results on the quality of the soils around the factories, metal ratios, geo-accumulation indexes, enrichment factors and pollution load indices of the media were calculated.^45^,^46^,^47^

#### Metal ratios

Metal ratios are often used to ascertain whether there has been enrichment or depletion relative to a background determined either from statistical methods (calculated background) or from prescribed guidelines (average crustal values) in the case of soil.^48^ For the present study, both methods were used to determine the background for the metal studied in the soil.^48^,^49^,^50^ The various metal ratios were then calculated relative to these established backgrounds (*Table 4*). The calculated background of any metal is dependent on the actual values measured from the study, while the average crustal values were derived from the averaging of a plethora of values of crustal materials measured in different parts of the world.^48^ The calculated background allows for discrimination of level of impact in areas that would ordinarily have indicated a polluted status for virtually all of the samples.

**Table 4:**
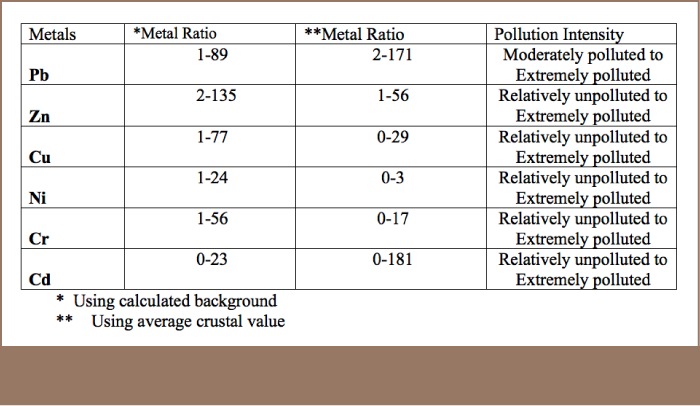
Summary of the Calculated Metal Ratios for Soils from the Study Area

**Table 5:**
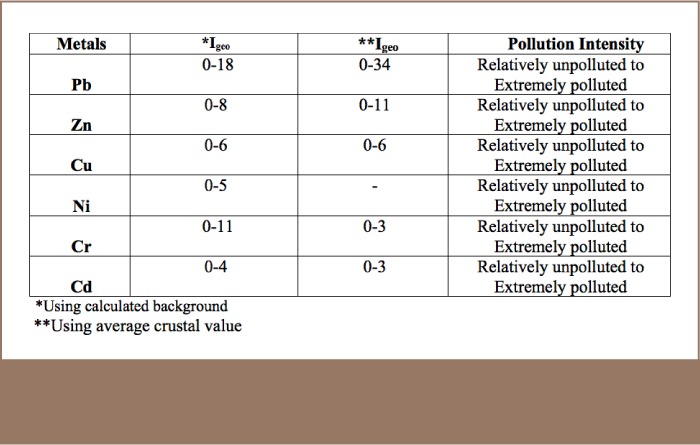
Summary of the Calculated Geo-accumulations (I_geo_) of Metals from Soil in the Study Area

The evaluated metal ratios ranged from less than 1.0 to several hundred folds greater, with the majority greater than (*Table 4*). This is a clear indication that the activities of the factories have led to localized elevated levels of enrichment of Pb, Zn, Cu, Cr and Cd and minimal enrichment of Ni relative to the various established backgrounds.

#### Geo-accumulation index

The geo-accumulation (I_geo_) index for the soil samples was also determined (*Supplemental Material 1*).^51^,^52^ The calculated I_geo_ revealed Pb, Zn, Cu and Cr as the main metals impacting soil quality in the immediate environment of the MRFs.

#### Contamination factor (CF)

Contamination factor is the ratio between an element at a location and the same element at a background site, or reference element (Al in this case) or an established criterion for that metal.^53^ The limitation of this calculation is that it does not consider the lithogenic input of the element of interest.^54^ The contamination factors calculated showed that Ni, Cr and Cu do not currently appear to be pollutants in the study area. The predominant contaminant metals were Pb and Zn in all of the study media (soil and PM). Soil was found to have several folds higher concentrations of the metals compared to PM (*Table 6*) This could be linked to prolonged settlement of the elements from the atmosphere (as aerosols) back into the soils.

**Table 6:**
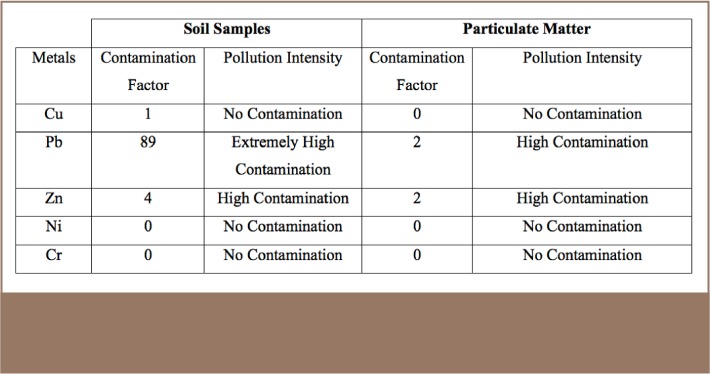
Summary of Contamination Factors for Soil and Particulate Matter Samples

#### Metal Relationship and Sourcing

All metals exhibited positive correlations with each other in the results for the studied soil (*Table 6*), an indication of the close relationship among the metals and the fact that they all originated from a similar source, the MRFs. However, for the R-mode factor analysis, Pb was more prominent in Factor 3, indicating that in addition to resulting from metal recycling activities, Pb may also have originated from the combustion of heavy fuels such as diesels used by vehicles and power generators in the area (*Tables 7, 8 and 9, Supplemental Material 4*).

**Table 7:**
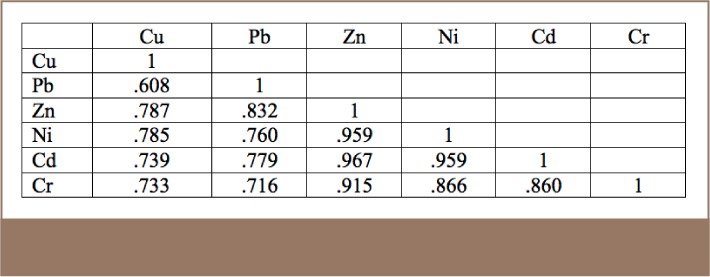
Correlation Matrix for the Results of the Soils Analysis

**Table 8:**
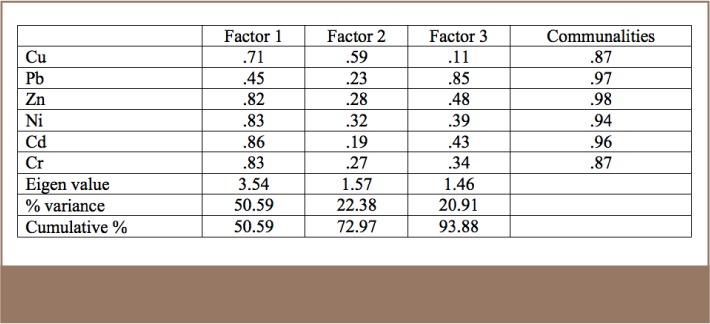
R-mode Factor Analysis of the Metal Results for Soil

**Table 9:**
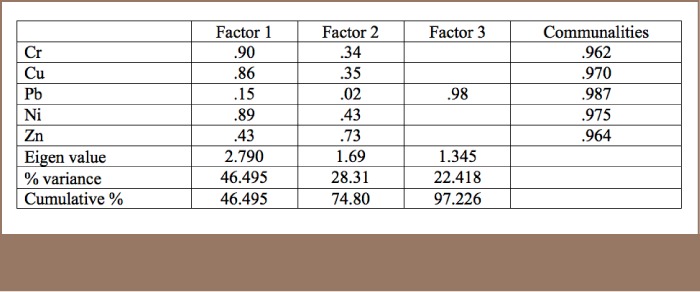
R-mode Factor Analysis of the Metal Results for Particulate Matter

## Conclusions

The present study demonstrated considerable enrichment in the levels of Pb, Cu, Zn, Ni, Cr and Cd in the soil samples, slags and PM collected from the study area. The sources of these enrichments in the soil, slags and PM can be attributed to the activities of the MRFs.

The results of the present study show that the metal recycling factories have negatively impacted the environment and have led to increased levels of deleterious metals in the soil and PM around the factories. The impacts on the inhabitants of the area have not yet been studied, and it is therefore very important to undertake a comprehensive environmental audit of these factories as well as to determine the health effects on the local inhabitants.
