# Supplementary material for: Evaluation of Pollution of Soils and Particulate Matter Around Metal Recycling Factories in Southwestern Nigeria
Source: J Health Pollut. 2018 Mar 12;8(17):20–30. doi: 10.5696/2156-9614-8.17.20 (PMC6221438; doi:10.5696/2156-9614-8.17.20)
Supplement: Supplementary file 1 [file i2156-9614-8-17-20.s1.docx]

**Supplemental Material 1**

**Metal distributions in the sampled media**


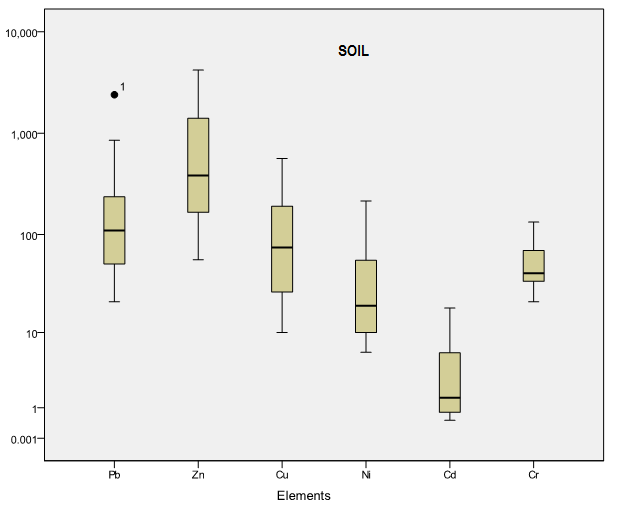


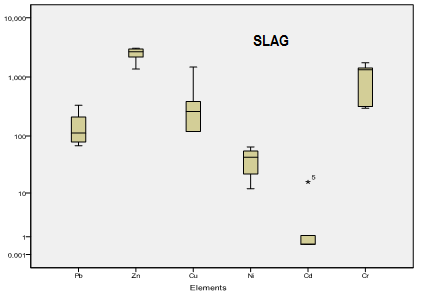


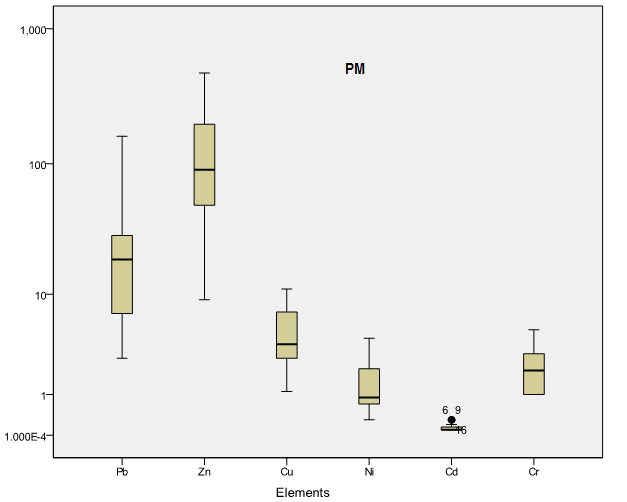


**Supplemental Material 2**

**Ternary compositional plots of selected metal oxides within individual mineral particles of particulate matter, as obtained from EDS spectra**


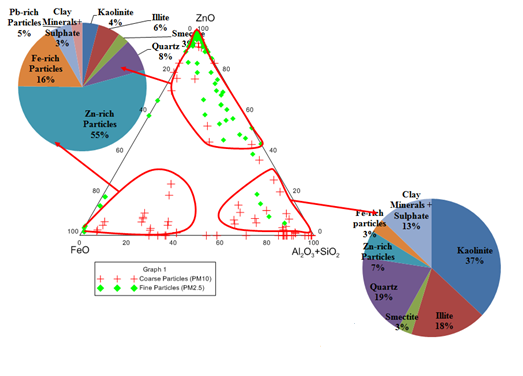


**Supplemental Material 3**

**Typical diffractogram of a soil sample from the study area**


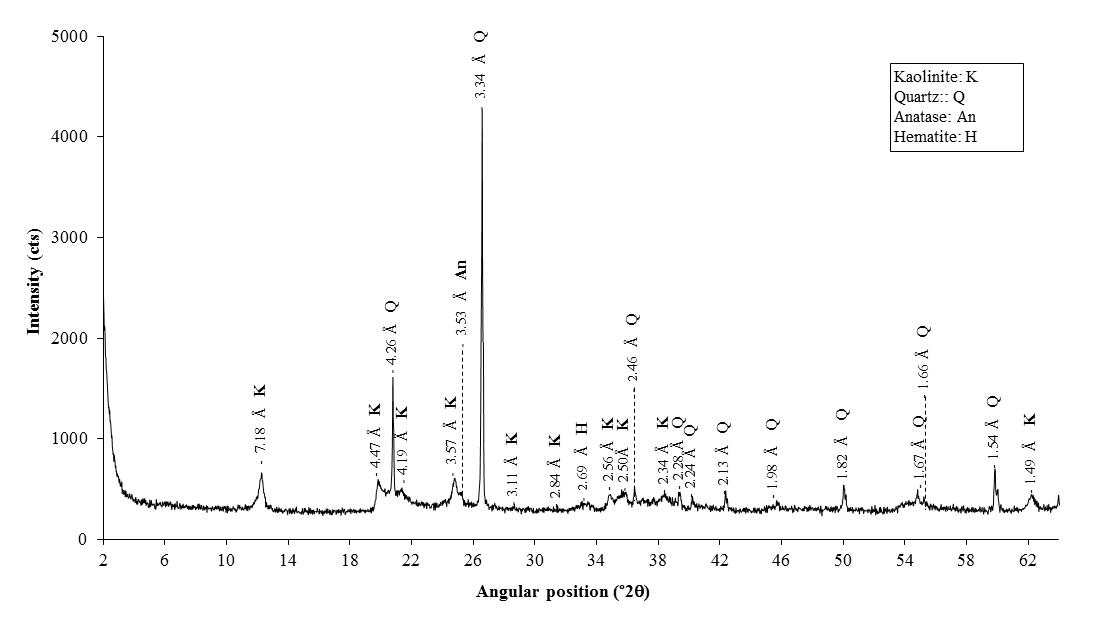


**Supplemental Material 4**

**
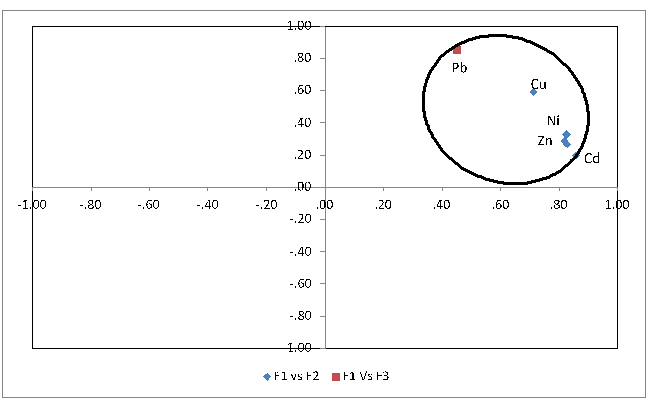
**

**Factor Plot of Soil (F1 vs F2/F3)**

**Factor Plot of PM_10_ (F1 vs F2/F3)**
